# Physicochemical Characterization and Evaluation of the Binding Effect of *Acacia etbaica* Schweinf Gum in Granule and Tablet Formulations

**DOI:** 10.1155/2021/5571507

**Published:** 2021-10-14

**Authors:** Kidan Haily Desta, Ebisa Tadese, Fantahun Molla

**Affiliations:** ^1^Department of Pharmacy, College of Medicine and Health Sciences, Wollo University, Ethiopia; ^2^Department of Pharmaceutics, School of Pharmacy, College of Health Sciences, Mekelle University, Ethiopia

## Abstract

This study is aimed at evaluating the binding effect of *Acacia etbaica* gum in granule and tablet formulations using paracetamol as a model drug. Some physicochemical properties of the purified gum such as pH, the presence of tannin and dextrin, solubility, viscosity, loss on drying, total ash value, water solubility index, swelling power, moisture sorption, and powder flow properties were investigated. Paracetamol granules were prepared using wet granulation method at 2%, 4%, 6%, and 8% *w*/*w* of the *Acacia etbaica* gum and compared with granules prepared with reference binders (PVP K-30 and Acacia BP) in similar concentrations. The granules were characterized for bulk and tapped densities, compressibility index and Hausner ratio, angle of repose, flow rate, and friability. Finally, the prepared granules were compressed into tablets and evaluated for different tablet characteristics: weight uniformity, thickness, diameter, crushing strength, tensile strength, friability, disintegration time, and *in vitro* release profile. The physicochemical characterization revealed that tannins and dextrin are absent in the gum, and the gum has acidic pH. Both the moisture content and total ash values were within the official limits. Furthermore, the gum was found to be soluble in cold and hot water but insoluble in organic solvent and exhibited a shear thickening viscosity profile and excellent flow properties with excellent compressibility. The granules prepared with the gum of *Acacia etbaica* and reference binders showed good particle size distribution and excellent flow and compressibility properties. All the prepared tablets passed pharmacopeial specifications with respect to their uniformity of weight, thickness, and disintegration time. Tablets formulated with *Acacia etbaica* gum and acacia BP meet the compendial specification for friability at binder concentrations more than 2%. Drug release properties of all the batches formulated with *Acacia etbaica*, PVP, and acacia BP complied with the pharmacopeial specification. It can be concluded that the gum of *Acacia etbaica* could be explored as an alternative excipient for its binder effect in granule and tablet formulations.

## 1. Introduction

Gums are pathological products formed following injury to the plant or due to unfavorable conditions such as drought and breakdown of cell walls (extracellular formation; gummosis) [1].

Gums are used as pharmaceutical excipients such as binding agents in tablet formulation. Binding agents are useful in achieving various tablet mechanical strength and drug release properties for different pharmaceutical purposes [2, 3].

In Ethiopia, more than 40 indigenous species of the genus Acacia have been identified, and about 17 of them are gum and gum resins bearing species. Most of the gum and resin bearing are naturally grown under arid, warm, and rugged topographic conditions in almost all regions of Tigray, Amhara, Oromia, Gambella, Somalia, Benshangul, Southern Region, and Afar in order of decreasing production area. *Acacia etbaica* is a member of *Fabaceae* family. It is a tree or shrub of 2.5-12 m tall ([4–6]).

The gum acacia obtained from different species of acacia have been proposed for various potential uses in pharmaceutical industry, such as stabilizer in emulsions and suspensions, binder in tablet formation, sustained drug release properties, and coating of medications [7–11]. However, no work has been reported so far on the use of gums of *Acacia etbaica.* Therefore, this study is aimed at characterizing the physicochemical properties of *Acacia etbaica* gum and evaluating its suitability as a binding agent in granules and tablet formulations in comparison with two reference binders, acacia BP and PVP, using paracetamol as a model drug. Paracetamol is a sparingly soluble drug with poor compression properties, which requires a binding agent among other excipients to form satisfactorily strong tablets [12].

## 2. Materials and Methods

### 2.1. Materials

The gum of *Acacia etbaica* was collected from Klte Awulaelo district, Eastern Zone of Tigray region; paracetamol (batch no. 1706003, HEDEI JIHENG, Pharmaceuticals, Germany), PVP K-30 (BOAINKY, Pharmaceutical Ltd., India), and Acacia BP (batch no. 242, BOAINKY, Pharmaceutical Ltd., India) were kindly donated from Addis Pharmaceutical Factory Sh. Co. Lactose, magnesium stearate, potassium dihydrogen phosphate, sodium hydroxide, sodium chloride, sodium hydrogen phosphate dehydrate, acetone, HCl (LOBA CHEMIE PVT. LTD, England), maize starch (DBEBATS, India), talc (SISCO LAB, India), ethanol (Batch no. 026193, Fine Chemical General Trading, Ethiopia), and chloroform (CHEMIE PVT. LTD., England) were purchased from local market. All the chemicals were analytical grade and used as received.

## 3. Methods

### 3.1. Purification of the Gum

The gum of *Acacia etbaica* size was reduced using an electrical grinder (NM-8300, Japan), dissolved in distilled water in 1 : 2 proportions, and filtered through a muslin cloth. Then, to the filtrate, 70% *v*/*v* absolute ethanol was added to precipitate the gum. The precipitated gum was then washed and dried in an oven (Beschickug 100-800, Germany) at 50°C for 18 hr. Finally, the dried gum was powdered to fine particles, sieved through a 180 *μ*m sieve (FRTSCH, 04003043, GERMANY) and stored in an air tight container [7].

### 3.2. Physicochemical Characterization of *Acacia etbaica* Gum

#### 3.2.1. Test for Tannins

Ferric chloride TS (0.1 mL) was added to 10 mL (10% *w*/*v*) of gum solution, formation of a dark blue color indicate the presence of tannins [13].

#### 3.2.2. Presence of Starch or Dextrin

A 10 mL quantity of gum (10% *w*/*v*) was boiled and cooled. Then, 0.1 mL iodine TS was added, and formation of blue or reddish-brown colour indicates the presence of dextrin [13].

#### 3.2.3. Relative Solubility Test

The gum powder (1 g) was added to 10 mL of cold and hot distilled water, acetone, chloroform, and ethanol. After 12 h, 5 mL of the clear supernatant was taken into small preweighed evaporating dishes and heated to dryness over a thermostatic water bath (HH-54, India) at 50°C for organic solvents and in an oven (Beschickug 100-800, Germany) at 105°C for 2 h for distilled water. The weights of the dried residue with reference to the volume of the solutions were determined using analytical balance (OHAUS, AR 3130, China) and expressed as the percentage solubility of the gum in the solvents [14].

#### 3.2.4. Water Solubility Index and Swelling Power

Water solubility index and swelling power of the gum was determined using the method described by Torruco-Uco and Betancur-Ancona [15]. First, 0.5 g of gum powder was weighed directly into centrifuge tubes, and 10 mL of distilled water was added to each tube. The tubes were kept in a thermostatically controlled water bath (HH-5454, India) at 25°C, 37°C, 55°C, 65°C, and 75°C for 30 minutes with frequent mixing in 2-minute intervals. The tubes were cooled and centrifuged (CENTRIFUGE, PLC-03, U.S.A) at 3000 rpm for 15 minutes. The supernatant was removed, and the sediment weight (Ws) was determined for each tube. The supernatant was dried to constant weight (W1) in an oven (Beschickug, 100-800, Germany) at 105°C for 12 hr. The water solubility index (WSI) and swelling power (SP) of the gum were calculated using Equations (1) and (2), respectively.

(1) $$\begin{array}{c} \text{WSI}=\frac{\text{W}1\times 100\%}{0.5}, \end{array}$$

(2) $$\begin{array}{c} \text{Sp}=\frac{Ws\times 100}{0.5\left(100-\text{WSI}\right)}, \end{array}$$

where WSI is the water solubility index, W1 is constant weight, Sp is swelling power, and Ws is sediment weight.

### 3.3. Viscosity of *Acacia etbaica* Gum

Viscometer (BROOKFIELD, RVDVE, U.S.A) was used to determine the effects of gum concentration and shear rate on apparent viscosity of the gum solution. To determine the effect of gum concentration on viscosity, different concentrations (5, 10, 20, and 30% *w*/*v*) of *Acacia etbaica* gum was dissolved in 250 mL of distilled water with continuous stirring. Then, viscosity measurements were conducted in triplicate at shear rate of 30 rpm. The effect of shear rate on apparent viscosity was determined by preparing 10% *w*/*v* of gum solution in 250 mL of distilled water with continuous stirring. Then, viscosity measurements were made at 30, 50, 60, and 100 rpm. All the measurements were carried out at room temperature [14].

### 3.4. Determination of the pH of Gum

The pH of the gum mucilage (5% *w*/*v*) was determined using a wagtech pH meter (AD 8000, Japan). The pH meter was set to neutral at a room temperature of 25°C, electrode was immersed into the gum mucilage, and the reading on the pH meter was recorded.

### 3.5. Loss on Drying

A 1.0 g quantity of the gum was transferred into a preweighed petri dish, then it was dried in an oven (Beschickug, 100-800, Germany) at 105°C until a constant weight was obtained. The loss on drying was determined as the ratio of the weight of moisture lost to the weight of sample multiplied by 100 [13].

### 3.6. Total Ash Determination

A 2 g sample of powder was weighed in a preweighed ashing crucible, followed by heating in a furnace (CARBOLITE, CWF 12/5, United Kingdom) at 450°C for 8 h. The sample was removed and kept in a desiccator, and weighed total ash values in the samples were calculated using

(3) $$\begin{array}{c} \text{Total ash}\left(\%\right)=\frac{m2-m1}{m}\times 100, \end{array}$$

where *m*1 is the mass of the ashing crucible, *m*2 is the mass of crucible plus ash, and *m* is the mass of the sample [13].

### 3.7. Moisture Sorption Study

Moisture sorption pattern was studied at different relative humidity. Pyrex desiccators containing distilled water, saturated solution of NaCl, and appropriate concentrations of NaOH (40, 31.58, and 24.66%) were prepared to obtain 100, 75.6, 60, 40, and 20% relative humidity (RH), respectively, and stored at room temperature. Two grams of oven predried, at 120°C for 4 h, *Acacia etbaica* gum powder was spread evenly on each petri dish and transferred to desiccators. Samples were equilibrated for four weeks at room temperature. The weights were recorded, and moisture uptake of each sample was calculated as the weight difference of the gum before and after equilibrium [16].

### 3.8. Determination of Density and Density-Related Properties

Thirty grams of the gum powder was introduced into 250 mL measuring cylinder, the volume occupied by the powder was noted, and the bulk density (*p*_*B*_) was calculated using

(4) $$\begin{array}{c} \text{Bulk density }\left(pB\right)=\frac{m}{VB}, \end{array}$$

where *m* is the mass of sample (in g) and *V*_*B*_ is the bulk volume of the sample powder (in mL).

To determine tapped density, the bulk in the cylinder was tapped using tapped densitometer (ERWEKA, SVM 223, Germany) at a rate of 250 taps/min. The volume occupied by the gum powder was recorded, and the tapped density (*P*_*T*_) was calculated using Equation (5) [13].

(5) $$\begin{array}{c} \text{Tapped density}\left(pT\right)=\frac{m}{VT}, \end{array}$$

where *m* is the mass of the sample (ing) and *V*_*T*_ is tapped volume of the sample (in mL).

Hausner ratio and Carr's index were calculated from bulk and tapped densities using Equations (6) and (7), respectively [13].

(6) $$\begin{array}{c} \text{Hausner Ratio}=\left(\;\frac{\rho T}{\rho B}\;\right), \end{array}$$

(7) $$\begin{array}{c} \text{Car}{\text{r}}^{\prime }\text{s index}\left(\%\right)=\left(\frac{\rho T-\rho B}{\rho T}\right)\times 100, \end{array}$$

where *ρB* is the bulk density and *ρT* is the tapped density.

### 3.9. Flow Property of the Gum Powder

Flow rate and angle of repose of the gum was determined using the powder flowability tester (PHARMA TEST, PT GS4, Germany).

### 3.10. Drug-Excipient Compatibility Studies

Fourier transform infrared (FTIR) spectroscopy was used to study the gum and drug interaction. The pure gum, the drug alone, and a mixture of the gum and the drug were mixed separately with IR grade KBr disc using paraffin as a film forming agent. The film on the disk was scanned over a wave number of 4000-400 cm^−1^ in an IR spectrometer (SHIMADZU, IR prestige 21, Japan). The major peaks of IR spectra of paracetamol in the mixture was analyzed and compared with the IR spectra of paracetamol alone.

### 3.11. Acute Oral Toxicity Test

Acute toxicity study of the gum *Acacia etbaica* was carried out in female mice that had an average weight of 25 g and age of 6-8 weeks. They were housed in appropriate cages in appropriate environment, fed standard diet, and allowed free access to water until used. The mice were divided into experimental and control groups of five mice each. The mice were fasted overnight, and the experimental groups received 5000 mg/kg of *Acacia etbaica* gum in distilled water, while the control group received 33.3 mL/kg of distilled water orally. The animals were observed for behavioral changes for the following 4 hrs and mortality for the next two weeks [17].

### 3.12. Preparation of Granules

Paracetamol granules were prepared using different concentrations (2, 4, 6, and 8% *w*/*w*), each of *Acacia etbaica* gum, PVP K-30, and Acacia BP as binders using the wet granulation technique. The desired quantities of paracetamol and lactose were dry mixed for 5 minutes. Then, the mixture was moistened with binder solution to produce granules containing various concentrations of binders. Wet massing was continued for 5 minutes, then granulated manually by passing through a 1400 *μ*m sieve and dried in a hot air oven (Beschickug 100-800, Germany) at 50°C for 18 h. The dried granules were passed through a 1000 *μ*m sieve [14].

### 3.13. Characterization of Granules

#### 3.13.1. Determination of Densities, Density-Related Properties, Flow Rate, and Angle of Repose

Granules were characterized for bulk and tapped densities, density-related properties, flow rate, and angle of repose as per the procedures described above for the gum powder.

#### 3.13.2. Determination of Granule Friability

Ten grams from each formulation of granule with larger than 315 *μ*m size were put into a Friabilator chamber (PHARMA TEST, PTF 10E, Germany) and allowed to revolve at 25 rpm for 4 minutes. The granules were then sieved using a 315 *μ*m sieve, and percent loss was calculated as friability.

#### 3.13.3. Preparation of Paracetamol Tablets

Different formulations of paracetamol tablets were prepared with the gum of *Acacia etbaica*, PVP K-30, and Acacia BP as shown in Table 1. The paracetamol granules were blended with maize starch, talc, and magnesium stearate for 5 minutes. The blended granules were compressed into tablets at a fixed compression force of 15 KN using tablet machine (RIVA S. A, MII 195, Buenos Aires, Argentina) which was fitted with 10 mm diameter flat-faced punches. The die volume was adjusted to get a target weight of 400 mg paracetamol tablet.

### 3.14. Evaluation of Paracetamol Tablets

#### 3.14.1. Weight Uniformity, Thickness, and Diameter of Tablets

Twenty tablets were randomly selected from each batch and assessed gravimetrically on individual basis using an analytical balance (OHAUS, AR 3130, China). The mean weight and standard deviation were calculated. The thickness and diameter of the tablets were measured using a tablet hardness tester (PHARMA TEST, PTB311E, Germany).

### 3.15. Crushing Strength

The force required to break 20 tablets was determined by the application of a diametrical force using a hardness tester (PHARMA TEST, PTB311E. Germany).

### 3.16. Tensile Strength

The tensile strengths (*σ*) of the tablets were calculated using the data obtained from crushing strength, diameter, and thickness of the tablet using

(8) $$\begin{array}{c} \sigma =\frac{2F}{\pi dt}, \end{array}$$

where *σ* is the tensile strength of the tablet (Nm^−2^), *F* is the crushing strength (N), *d* is the tablet diameter (m), and *t* is the thickness (m).

### 3.17. Friability Testing

Ten intact tablets were randomly selected, dedusted, and weighed. The tablets were placed in Friabilator (PHARMA TEST, PTF 10E, Germany) and subjected to tumbling actions at 25 rpm for 4 minutes. Finally, the tablets were once again dedusted and reweighed to determine the percentage loss of weight.

### 3.18. Disintegration Tests

The disintegration test was done using six tablets from each batch in distilled water at 37 ± 2°C using a disintegration apparatus (PHARMA TEST, PTZS 230, Germany). The disintegration time was taken to be the time at which no granule of any tablet was left on the meshes of the apparatus [13].

### 3.19. *In Vitro* Drug Release Studies

The release profile of paracetamol from tablets was determined using USP type II dissolution apparatus (PHARMA TEST, PTWS 20D, Germany) at paddle rotation speed of 50 rpm. The dissolution medium was 900 mL of phosphate buffer of pH 5.8 maintained at a temperature of 37 ± 0.5°C. In all experiments, 5 mL of sample was withdrawn at 5, 10, 15, 20, 30, 45, and 60 min. Each withdrawn sample was replaced with an equal volume of fresh medium at the same temperature to maintain sink condition. Absorbance of the samples was measured using UV/visible spectrophotometer at 243 nm after appropriate dilution with phosphate buffer of pH 5.8. Percentage cumulative drug release was calculated using the equation obtained from a standard curve.

### 3.20. Statistical Analysis

Microsoft Excel and Origin® 8 software (OriginLab Corporation, MA, USA) were used for the statistical analysis. One-way analysis of variance (ANOVA) was used to compare the effect of binder concentration on granule and tablet mechanical and release properties. Tukey multiple comparison test was used to compare the differences between the properties of the different batches of granules and tablets. At 95% confidence interval, *P* values less than or equal to 0.05 were considered significant. All the values are given as mean and standard deviation.

## 4. Results and Discussions

### 4.1. Physicochemical Characteristics of the Gum

As indicated in Table 2, the pH of the gum solution (5% *w*/*v*) at a temperature of 25°C was 5.67 ± 0.06. This indicates that the gum is weakly acidic in nature. This is attributed to the fact that gums are generally macromolecular acids due to the presence of uronic acids in their structure [18].

The moisture content and total ash values of *Acacia etbaica* gum were 12 ± 0.28% and 3 ± 0.70%, respectively, which is within the pharmacopeial specification. The pharmacopeial set for the former is maximum of 15%, and the latter is maximum of 4% for acacia gum [13].

The solution of gum of *Acacia etbaica* did not show any bluish or reddish color upon the addition of iodine TS, which confirms the absence of starch or dextrin in the purified gum. Moreover, there was no blackish precipitate when ferric chloride TS was added which indicate the gum does not contain tannins. This is similar with the results of *Acacia polyacantha* [7].

### 4.2. Relative Solubility of the Gum

The solubility of *Acacia etbaica* gum in different solvents is presented in Table 3. Accordingly, the gum is soluble in hot and cold water and insoluble in organic solvents. The solubility of *Acacia etbaica* gum in aqueous solvent may be attributed to the branched nature of the polymer, which has been reported to be more soluble compared to the linear components. Any structural feature such as branching structure and charged group (carboxylate group, sulfate and phosphate groups) that hinders the intermolecular association leads to higher solubility. Structural characters such as linear chain and large molecular weight that promote the intermolecular association result in a poor solubility [19].

The solubility of the gum in hot water is higher than the corresponding solubility in cold water, since solubility increases with increase in temperature for most cases [20].

### 4.3. Water Solubility Index and Swelling Power

The solubility and swelling power of the gum are shown in Table 4. The solubility index and swelling power of the gum were generally increased with temperature. This may be due to disruption of intermolecular hydrogen bonds which maintain the structural integrity of the gum with increased temperature [21].

### 4.4. Moisture Sorption Properties

**Figure 1:**
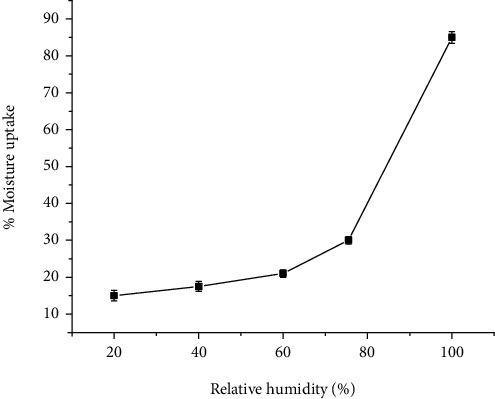
Moisture sorption pattern of *Acacia etbaica* gum.

The moisture sorption characteristic of pharmaceutical excipients is imperative since most of the physicochemical, functional, and stability properties are affected by moisture. Moisture sorption study is important for the selection of suitable packaging materials and storage area of excipients [22].  Figure 1 shows the moisture sorption profile of *Acacia etbaica* gum equilibrated at various humidity levels. Moisture sorption increased generally with the RH. This is implying that the gum is hygroscopic at higher relative humidity values.

### 4.5. Viscosity

**Figure 2:**
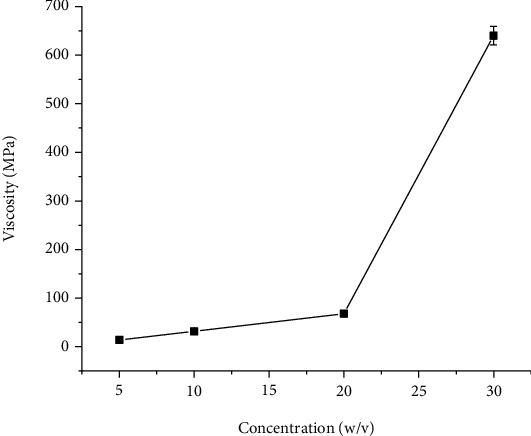
Apparent viscosity of *Acacia etbaica* gum solution at different gum concentrations measured at 30 rpm.

**Figure 3:**
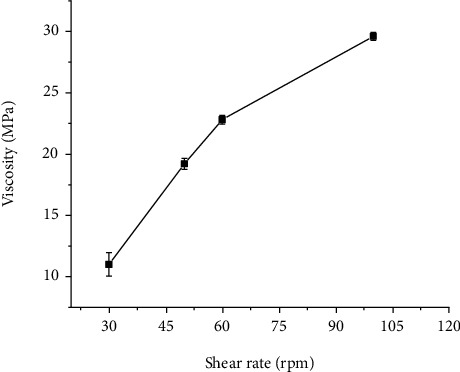
Apparent viscosity of 10% *w*/*vAcacia etbaica* gum solution at different shear rates.

The application of plant gums is due to water-holding capacity to form a gel structure; this enhance the stability of formulation. Viscosity gives information about the molecular structure and the utilization of the gum [23]. As shown in Figure 2, the viscosity of the gum solution increased upon increasing the mucilage concentration. The higher viscosity obtained at higher concentrations of gum may be due to increased intermolecular interaction between the gum molecules and reduction in gum-solvent interaction [20]. The effect of shear rate on apparent viscosities of the solution is presented in Figure 3. The viscosities of the gum increased with increase in the shear rate. This shows the gum has a dilatant or shear-thickening behavior.

### 4.6. Flow Properties of the Gum Powder

Bulk and tapped densities give an insight on the packing arrangement of the particles and the compaction profile of a material. They can provide information on the flowability of powders and use to calculate Carr's index and Hausner's ratio, which are a measure of the compressibility and flowability of a powder. The angle of repose could be used as a quantitative measure of the cohesiveness or the tendency of powder to flow [24]. As shown in Table 5, the values of Carr's index, Hausner's ratio, and angle of repose of the gum indicate that the gum has excellent flow properties with excellent compressibility. This minimizes the modification of formulations for the improvement of the flow properties of the gum when it is used as an excipient in a pharmaceutical formulation [25].

### 4.7. Drug-Excipient Compatibility Study

**Figure 4:**
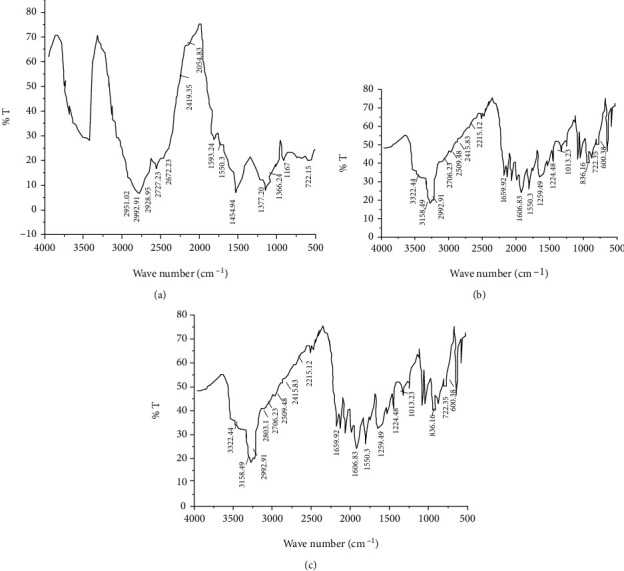
FTIR spectrum of *Acacia etbaica* gum (a), paracetamol standard (b), and mixture of *Acacia etbaica* gum and paracetamol (c).

Figures 4(a)–4(c) show the FTIR spectra of *Acacia etbaica* gum, paracetamol standard, and mixture of *Acacia etbaica* gum and paracetamol. As shown in Figure 4(a), the characteristic peaks of paracetamol are O–H stretching at 3322.44 cm^−1^, N–H stretching at 3158.41 cm^−1^, C=O stretching of the carboxyl ion at 1655.92 cm^−1^, C–N–H group at 1259.54 cm^−1^, and paradisubstituted aromatic ring at 836.16 cm^−1^ which were observed confirming the purity of the drug as per established standards. As shown in Figure 4(c), the O–H stretching band of paracetamol at 3322.44 cm^−1^ was also observed in the spectrum of its mixture with the gum. The absorption band N-H stretching at 3159.18 cm^−1^ and C=O stretching at 1655.92 cm^−1^ appeared in the spectrum of the physical mixture of paracetamol and *Acacia etbaica*. The C–H stretching band of paracetamol at 3000-2900 cm^−1^ was merged with C–H stretching of the *Acacia etbaica.* The C–N–H group was observed at 1259.54 cm^−1^ and paradisubstituted aromatic ring at 836.16 cm^−1^. This indicated that there were no physical and chemical interactions between the gum and paracetamol as there were no distinct changes in the available peaks with their corresponding wave numbers.

### 4.8. Acute Oral Toxicity

The findings of this study showed that the gum has no sign of acute toxicity. This was confirmed since the mice did not show behavioral changes like loss of appetite, hair erection, lacrimation, convulsions, salivation, and diarrhea for the next 4 hrs after administration of the gum dose of 5 g/kg body weight. In addition, no death was recorded in the next two days and even the following two weeks.

### 4.9. Evaluation of Granules

#### 4.9.1. Density and Density-Related Properties

Densities of granules influence flowability and compressibility of the granules which can affect the quality of tablets. Granules having higher bulk density require relatively lower die fill volume than those having small bulk density. Density and related properties of the paracetamol granules prepared using *Acacia etbaica* gum, PVP k-30, and Acacia BP are presented in Table 6. The bulk and tapped densities of the granules prepared at all concentration and types of binder were decreased with increasing concentrations of the binders. This could be attributed to the increase in the proportion of larger granules with increasing binder concentrations. The granules occupy larger volumes making the bulk density value lower than smaller granules which occupy smaller bulk volumes [26]. At 2% and 4% binder concentration, granules prepared with *Acacia etbaica* had significantly lower bulk and tapped density than that of PVP and Acacia BP (*P* < 0.05). However, there was no significant difference in bulk and tapped density in all types of binders prepared with 6% and 8% binder concentrations (*P* > 0.05). Table 6 also shows that all the formulations have Carr's index values below 15% implying the granules have excellent flow property. Hausner's ratios were also observed to be less than 1.25, which also confirmed excellent flow property of the granules. Excellent flow property of the granule is necessary to ensure efficient mixing and acceptable weight uniformity in compressed tablets [27].

#### 4.9.2. Flow Rate and Angle of Repose

Table 7 presents granule flow rate and angle of repose. As shown in the table, the angle of repose was decreased as the concentration of binder increased. This may be attributed to the reduced cohesive forces of the larger granules formed at higher binder concentration [28]. The angle of repose was below 30° for all the formulations, indicating the free flowing property of granules. This excellent flowability of granules is important for acceptable weight variation and content uniformity of tablets [29].

#### 4.9.3. Granule Friability

**Figure 5:**
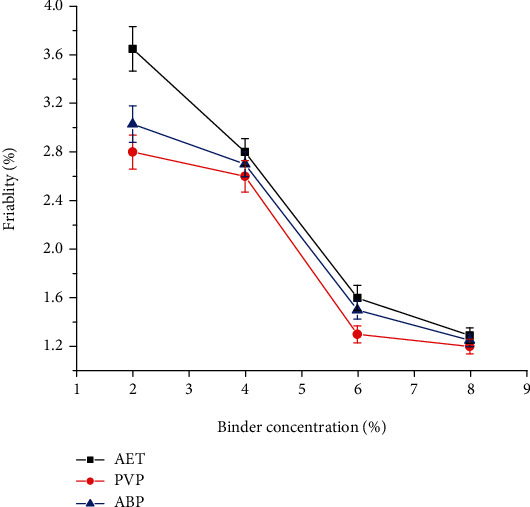
Friability of granules prepared with different concentrations of *Acacia etbaica* gum, PVP k-30, and Acacia BP.

Figure 5 shows the friability value of the different batches of granules. Accordingly, the friability values of the granules were decreased with increased binder concentration. This is due to the fact that as the concentration of binder increased, more bonding is attained so that the strength of the granules increased and became resistant to abrasion during the test. The percent friability of paracetamol granules formulated at 2% of AET was significantly higher than that of PVP and ABP (*P* < 0.05). There was no significant difference in friability values between all type of binders at 4%, 6%, and 8% binder concentration (*P* > 0.05).

### 4.10. Tablet Properties

#### 4.10.1. Weight Uniformity, Thickness, and Diameter

Weight uniformity, thickness, and diameter of tablets are presented in Table 8. The weight of tablets prepared using different binders and at various concentrations met the compendial specification. The compendial specification for uniformity of weight states that for tablets weighing more than 250 mg, weights of more than two tablets should not deviate from the average weight of 20 tablets by more than 5% [13].

At 2% and 4% *w*/*w* binder concentration, thickness of tablet prepared with *Acacia etbaica* is significantly higher than acacia BP (*P* < 0.05). But, there was no significant difference in thickness among the tablets formulated with *Acacia etbaica* gum and PVP at 6 and 8% *w*/*w* binder concentration.

#### 4.10.2. Crushing Strength, Tensile Strength, and Friability

**Figure 6:**
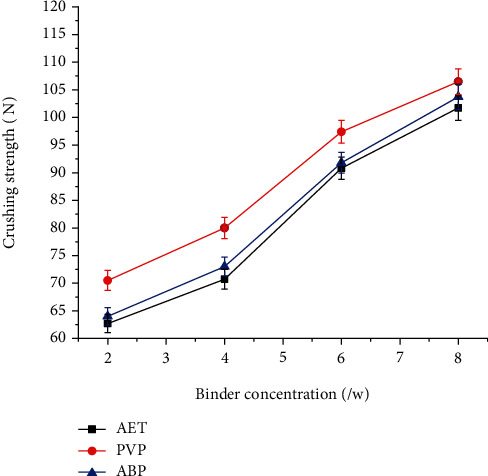
Effect of binder concentration on the crushing strength of paracetamol tablets prepared at different concentrations of *Acacia etbaica* (AET), polyvinylpyrrolidone (PVP), and Acacia BP (ABP).

Crushing strength of solid oral dosage forms provides a quantitative estimate of the internal bonding strength of the powder compact, which gives the tablet sufficient mechanical strength to maintain its internal structure and geometry under the applied external forces [30]. As shown in Figure 6, the crushing strength of tablets was increased as the binder concentration increased. The crushing strength of tablets prepared with 2%, 4%, and 6% of *Acacia etbaica* gum was significantly lower than that of PVP (*P* < 0.05), but almost similar to that of Acacia BP (*P* > 0.05). However, there was no significant difference in all binder types at 8% binder concentration.

**Figure 7:**
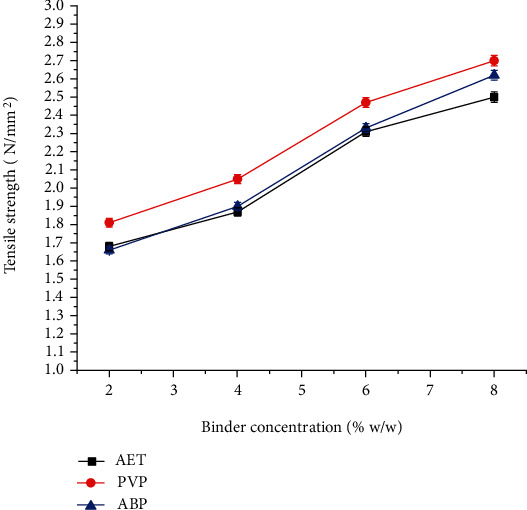
Effect of binder concentration on the tensile strength of paracetamol tablets prepared at different concentrations of *Acacia etbaica* (AET), polyvinylpyrrolidone (PVP), and Acacia BP (ABP).

The tensile strength of paracetamol tablets containing different concentrations of the *Acacia etbaica gum* and reference binders are presented in Figure 7. Accordingly, the tensile strength of tablets formulated with PVP was significantly higher than that of *Acacia etbaica* at all concentration and types of binder (*P* < 0.05). Nevertheless, there was no significant difference among Acacia BP and *Acacia etbaica* at 2%, 4%, and 6% binder concentration (*P* > 0.05).

**Figure 8:**
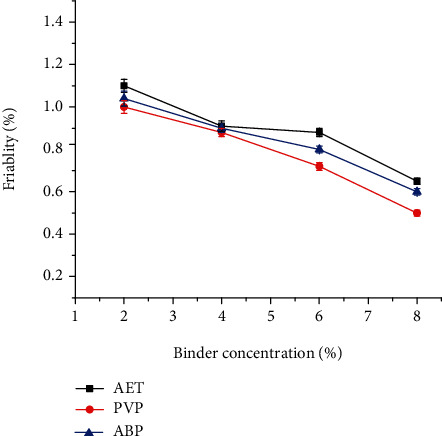
Effect of binder concentration on the friability of paracetamol tablets prepared at different concentrations of *Acacia etbaica* (AET), polyvinylpyrrolidone (PVP), and Acacia BP (ABP).

Friability testing is employed to determine the physical strength of compressed and uncoated tablets upon exposure to mechanical shock and attrition [31]. As shown in Figure 8, the friability of the tablets decreased as the concentration of the binder increased. This may be due to increase in binder concentration results in increased intragranular force during compression leads lower rate of chipping on abrasion [32].

Tablets formulated with *Acacia etbaica* gum and Acacia BP meets the compendial specification for friability at binder concentrations more than 2%. On the other hand, tablets with PVP meet friability tests (>1%) at all binder concentration.

### 4.11. Disintegration Time

**Figure 9:**
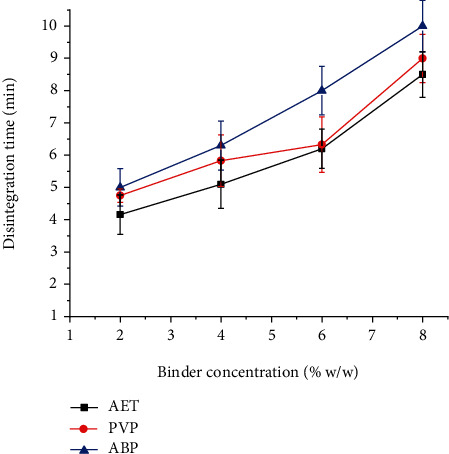
Effect of binder concentration on the disintegration time of paracetamol tablets prepared at different concentrations of *Acacia etbaica* (AET), polyvinylpyrrolidone (PVP), and Acacia BP (ABP).

Disintegration is a physical process related to the mechanical breakdown of a tablet into smaller particles. It represents the breakdown of interparticle interactions generated during tablet compaction of granulated particles of the active pharmaceutical ingredient and excipients [33]. As shown in Figure 9, the disintegration time of the tablets increased as the binder concentration increased. This may be due to increase in binder concentration results in increase in cohesion between the particles; therefore, more time is needed to separate the particles [34].

All the prepared tablets disintegrated within 10 minutes, which conformed to the British Phamacopeia [13] specification for disintegration that states uncoated tablets should disintegrate within 15 minutes. There was no significant difference in disintegration time among tablets formulated with 2% and 4% binder concentration of AET, PVP, and ABP (*P* > 0.05). However, the disintegration time of tablets formulated at 6% and 8% of ABP was significantly longer than that of PVP and AET (*P* < 0.05).

### 4.12. *In Vitro* Drug Release Studies

**Figure 10:**
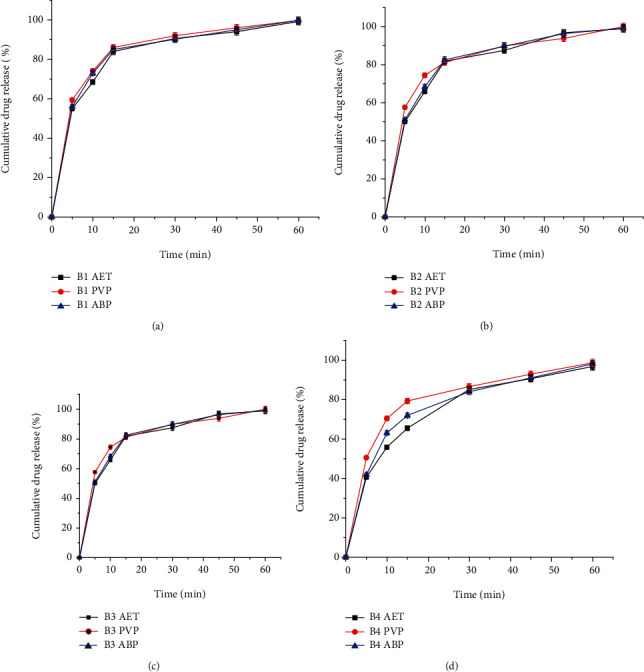
*In vitr*o release profile of paracetamol from tablets formulated with 2% (a), 4% (b), 6% (c), and 8% (d) binder concentrations of *Acacia etbaica* gum (AET), polyvinylpyrrolidone (PVP), and Acacia BP (ABP) (*n* = 6, mean ± SD).

*In vitro* drug release is used for measuring the time required for a given percentage of the drug substance in a tablet to dissolve under a specified set of conditions in an *in vitro* test. It is intended to provide information on physiological availability of the drug substance. Binders and granulating agents included in tablet formulation and other solid dosage forms can distinctly affect the dissolution of drugs [35]. Figures 10(a)–10(d) show the *in vitro* drug release profiles of the tablets prepared with different concentrations of binders. As the binder concentration increased, the release rate of paracetamol from the tablets generally decreased. This might be due to the higher bond strength of tablets with increased binder concentration that prolonged the dissolution time [36]. The USP 30/NF 25 [37] states that the quantity of drug released should not be less than 80% of the labeled amount of paracetamol in 30 minutes. All tablets formulated at various binder concentration of *Acacia etbaica* gum, polyvinylpyrrolidone, and Acacia BP meet the compendial specification. There was no significant difference in drug release profile among tablets formulated at 2%, 4%, and 6% binder concentration of AET, PVP, and ABP in 30 minutes (*P* > 0.05). However, the drug release rate of tablets formulated at 8% of PVP Figure 10(d) was significantly higher than that of AET and ABP (*P* < 0.05). This is due to the higher concentration of gums in the tablets forms a thicker and more viscous gel layer which appears a more strong barrier that decrease the drug release [38].

## 5. Conclusion

The physicochemical characterization of the *Acacia etbaica* gum revealed that tannin and dextrin are absent in the gum, and the moisture content and ash values were within the pharmacopeial limit. The gum was also found to be soluble in cold and hot water but insoluble in organic solvents and exhibited excellent flowability and compressibility. The oral acute toxicity study in mice showed that the gum is safe up to 5 g/kg body weight. The paracetamol granules were prepared by wet granulation technique using *Acacia etbaica* gum as binder acceptable flow properties and friability which were comparable to those prepared with reference binders. The tablets prepared with *Acacia etbaica* gum exhibited comparable physical and mechanical properties with respect to crushing strength, tensile strength, friability, uniformity of weight, disintegration time, and drug release profile with that of PVP and Acacia BP. Hence, it can be concluded that the gum of *Acacia etbaica* (Schweinf) can be positively explored as an alternative excipient for its binding effect in granule and tablet formulations.

## Figures and Tables

**Table 1:** Composition of the different formulations of paracetamol tablets prepared using gum of *Acacia etbaica* (AET), PVP K-30 (PVP), and Acacia BP (ABP) as binders.

Formulations	Ingredients (mg)								
	Paracetamol	AET	PVP K-30	Acacia BP	Lactose	Maize starch	Mg stearate	Talc	Total (mg)/tab
AET1	300	8	—	—	46	40	2	4	400
AET2	300	16	—	—	38	40	2	4	400
AET3	300	24	—	—	30	40	2	4	400
AET4	300	32	—	—	22	40	2	4	400
PVP1	300	—	8	—	46	40	2	4	400
PVP2	300	—	16	—	38	40	2	4	400
PVP3	300	—	24	—	30	40	2	4	400
PVP4	300	—	32	—	22	40	2	4	400
ABP1	300	—	—	8	46	40	2	4	400
ABP2	300	—	—	16	38	40	2	4	400
ABP3	300	—	—	24	30	40	2	4	400
ABP4	300	—	—	32	22	40	2	4	400

**Table 2:** Some common physicochemical properties of *Acacia etbaica* gum (*n* = 3, mean ± SD).

Parameter	Result
PH	5.67 ± 0.06
Loss on drying (%)	12 ± 0.28
Total ash value (%)	3 ± 0.70
Tannins	Absent
Dextrin	Absent

**Table 3:** Solubility of *Acacia etbaica* gum in different solvents (*n* = 3, mean ± SD).

Solvent	Solubility (g/mL)
Hot distilled water	0.046 ± 0.02
Cold distilled water	0.037 ± 0.03
Ethanol (96%)	0.008 ± 0.05
Acetone	0.006 ± 0.07
Chloroform	0.004 ± 0.02

**Table 4:** Water solubility index and swelling power of *Acacia etbaica* gum at different temperatures (*n* = 3, mean ± SD).

Temp (°C)	25	37	55	65	75
WSI (%)	8 ± 0.02	16 ± 0.74	24 ± 1.02	48 ± 0.81	56 ± 0.04
SP (ratio)	3.47 ± 0.05	4.76 ± 0.04	6.31 ± 0.02	10.76 ± 0.03	16 ± 0.06

**Table 5:** Flow properties of *Acacia etbaica* gum (*n* = 3, mean ± SD).

Variables	Values
Bulk density (g/mL)	0.74 ± 0.02
Tapped density (g/mL)	0.78 ± 0.04
Carr's index (%)	5.51 ± 0.03
Hausner's ratio	1.05 ± 0.01
Flow rate (g/sec)	12.5 ± 0.41
Angle of repose (degrees)	25.5 ± 0.76

**Table 6:** Bulk and tapped densities and density-related properties of paracetamol granules prepared at different concentrations of *Acacia etbaica* (AET), polyvinylpyrrolidone (PVP), and Acacia BP (ABP) (*n* = 3, mean (± SD)).

Formulation	Bulk density (g/mL)	Tapped density (g/mL)	Carr's index (%)	Hausner's ratio
AET1	0.45 (0.04)	0.48 (0.01)	6.25 (0.01)	1.07 (0.02)
AET2	0.44 (0.03)	0.47 (0.03)	4.34 (0.03)	1.07 (0.03)
AET3	0.43 (0.01)	0.45 (0.02)	4.44 (0.01)	1.04 (0.02)
AET4	0.41 (0.01)	0.43 (0.04)	4.65 (0.02)	1.04 (0.02)
PVP1	0.55 (0.02)	0.58 (0.06)	5.17 (0.02)	1.05 (0.04)
PVP 2	0.54 (0.03)	0.57 (0.05)	5.25 (0.04)	1.05 (0.02)
PVP 3	0.47 (0.04)	0.48 (0.02)	2.08 (0.01)	1.02 (0.02)
PVP 4	0.44 (0.07)	0.46 (0.03)	2.27 (0.02)	1.05 (0.03)
ABP 1	0.55 (0.06)	0.59 (0.04)	6.74 (0.03)	1.07 (0.02)
ABP 2	0.53 (0.05)	0.55 (0.05)	5.05 (0.02)	1.05 (0.03)
ABP 3	0.45 (0.01)	0.47 (0.01)	4.2 (0.01)	1.04 (0.01)
ABP 4	0.44 (0.02)	0.45 (0.01)	2.2 (0.02)	1.02 (0.02)

**Table 7:** Flow rate and angle of repose of paracetamol granules prepared at different concentrations of *Acacia etbaica* (AET), polyvinylpyrrolidone (PVP), and Acacia BP (ABP) (*n* = 3, mean ± SD).

	Flow rate (gm/sec)	Angle of repose (*Ө*)
AET1	16.6 ± 0.21	27.8 ± 0.74
AET 2	17.6 ± 0.46	27.2 ± 0.51
AET 3	19.35 ± 0.73	26.8 ± 1.02
AET 4	21.4 ± 0.54	26.6 ± 1.04
PVP1	17.6 ± 0.61	27.5 ± 0.83
PVP 2	18.75 ± 0.42	26.3 ± 0.68
PVP 3	20 ± 0.33	26.5 ± 1.03
PVP 4	21.07 ± 0.52	25.4 ± 0.57
ABP 1	17.1 ± 0.66	26.9 ± 1.06
ABP 2	18.75 ± 0.35	26.5 ± 0.92
ABP 3	20 ± 0.12	26.1 ± 0.63
ABP 4	20.6 ± 0.06	25.6 ± 0.71

**Table 8:** Weight and thickness of the different formulations of paracetamol tablets prepared at different concentrations of *Acacia etbaica* (AET), polyvinylpyrrolidone (PVP), and Acacia BP (ABP) (*n* = 20, mean ± SD).

Formulation	Weight (mg)	Thickness (mm)	Diameter (mm)
AET 1	396.43 ± 1.57	4.3 ± 0.09	9.95 ± 0.03
AET 2	400.1 ± 2.08	4.24 ± 0.12	9.97 ± 0.09
AET 3	403.8 ± 2.21	4.09 ± 0.06	9.98 ± 0.07
AET 4	397.9 ± 1.75	4.03 ± 0.07	9.99 ± 0.08
PVP1	398.7 ± 1.54	4.13 ± 0.13	9.96 ± 0.04
PVP 2	402.17 ± 2.23	4.16 ± 0.06	9.98 ± 0.06
PVP 3	404.52 ± 1.69	4.14 ± 0.12	9.98 ± 0.01
PVP 4	399.03 ± 1.9	4.07 ± 0.1	9.99 ± 0.02
ABP 1	397.3 ± 1.72	4.12 ± 0.03	9.96 ± 0.05
ABP 2	402.7 ± 2.27	4.16 ± 0.06	9.97 ± 0.04
ABP 3	401.2 ± 1.08	4.06 ± 0.11	9.99 ± 0.07
ABP 4	398.02 ± 1.94	4.08 ± 0.08	10.01 ± 0.08

## Data Availability

The data used to support the findings of this study are available from the corresponding author upon request.

## References

[1] Majekodunmi S. O., Makpe S. (2016). Development and evaluation of the binding properties of *Raphia hookeri* (Fam. Palmaceae) gum in pharmaceutical tablet formulations. *Journal of Pharmaceutical Research International*.

[2] Jain A., Radiya P., Wadekar R., Limaye S., Pawar C. (2014). Natural excipients-an alternative to synthetic excipients: a comprehensive review. *International Journal of Pharmaceutical and Medicinal Research*.

[3] Patil P. S. (2014). Natural excipients: uses of pharmaceutical formulations. *International Journal of PharmTech Research*.

[4] Belay Z., Vestberg M., Assefa F. (2013). Diversity and abundance of arbuscular mycorrhizal fungi associated with acacia trees from different land use systems in Ethiopia. *African Journal of Microbiology Research*.

[5] Orwa C., Mutua A., Kindt R., Jamnadass R., Anthony S. Agroforestree database: a tree reference and selection guide version 4.0. http://www.worldagroforestry.org/sites/treedbs/treedatabases.asp.

[6] Worku A., Lemenih M., Fetene M., Teketay D. (2011). Socio-economic importance of gum and resin resources in the dry woodlands of Borana, southern Ethiopia. *Forests, Trees and Livelihoods*.

[7] Aklilu T., Abdel-Rahman S. I., Gebre-Mariam T. (2002). Local gum of *Acacia polyacantha* as a binder in tablet formulations: effect on granule properties. *The Ethiopian Pharmaceutical Journal*.

[8] Brhane Y., Belete A., Gebre-Mariam T. (2014). Evaluation of local um of *Acacia polyacantha* as a suspending agent in metronidazole benzoate suspension formulations. *Ethiopian Pharmaceutical Journal*.

[9] Karmakar K. (2016). Application of natural gum as a binder in modern drug delivery. *Journal of Analytical & Pharmaceutical Research*.

[10] Mankala S. K., Nagamalli N. K., Raprla R., Kommula R. (2011). Preparation and characterization of mucoadhesive microcapsules of Gliclazide with natural gums. *Stamford Journal of Pharmaceutical Sciences*.

[11] Seleshi A., Belete A., Gebre-Mariam T. (2002). Evaluation of native and cross-linked Acacia Senegal (l.) Willd. var. Senegal and Acacia Senegal (l.) Willd. Var. Karensis gums as excipients for sustained release formulations I. Effects on granule properties. *The Ethiopian Pharmaceutical Journal*.

[12] Adetogun G. E., Alebiowu G. (2009). Properties of Delonix regia seed gum as a novel tablet binder. *Acta Poloniae Pharmaceutica - Drug Research*.

[13] British Pharmacopeia (2013). *The Pharmaceutical Press, Her Majesty’s Stationery Office, London*.

[14] Tadese E., Belete A., Gebre-Mariam T. (2014). Evaluation of the binding effect of local gum of *Boswellia Papyrifera* in paracetamol granules and tablet formulations. *The Ethiopian Pharmaceutical Journal*.

[15] Torrucouco J., Betancur-Ancona D. (2007). Physicochemical and functional properties of makal (*Xanthosoma yucatanensis*) starch. *Food Chemistry*.

[16] Olayemi O. J., Oyi A. R., Allagh T. S. (2008). Comparative evaluation of maize, rice and wheat starch powders as pharmaceutical excipients. *Nigerian Journal of Pharmaceutical Sciences*.

[17] Gebresamuel N., Gebre-Mariam T. (2012). Comparative physico-chemical characterization of the mucilages of two cactus pears (*Opuntia* spp.) obtained from Mekelle, Northern Ethiopia. *Journal of Biomaterials and Nanobiotechnology*.

[18] Bilal S., Mohammed-Dabo I. A., Dewu B. B. M., Momoh O. R., Abubakar S. (2015). Refining and characterisation of gum arabic using vacuum filtration method for application in oil and gas drilling fluid formulation. *Journal of Experimental Research*.

[19] Lima R. S. N., Lima J. R., Salis C. R., Moreira R. A. (2002). Cashew tree (*Anacardium occidentale* L.) exudates gum: a novel bioligand tool. *Biotechnology and Applied Biochemistry*.

[20] Ameh P. O. (2012). Physicochemical properties and rheological behaviour of *Ficus glumosa* gum in aqueous solution. *International Journal of Modern Chemistry*.

[21] Yagoub N., Nur A. O. (2013). The influence of thermal treatment on physical properties of guar gum. *International Journal of Innovations in Pharmaceutical Sciences*.

[22] Chukwu O. (2010). Moisture-sorption study of dried date fruits. *AU Journal of Technology*.

[23] Miao Q., Jiang H., Gao L. (2018). Rheological properties of five plant gums. *American Journal of Analytical Chemistry*.

[24] Sarangapani S., Rajappan M. (2012). Pharmacognostical and pharmaceutical characterisation of delonix regia - a novel matrix forming natural poymer. *International Journal of Pharmaceutics*.

[25] Biswajit D., Suvakanta D., Chandra C. R., Jashabir C., Saumendu D. R. (2014). Optimization and characterization of purified polysaccharide from Terminalia belarica gum as pharmaceutical excipient. *International Journal of Pharmaceutical Research & Allied Sciences*.

[26] Singh A. K., Shingala V. K., Selvam R. P., Sivakumar T. (2010). Evaluation of *Mangifera indica* gum as tablet binder. *International Journal of PharmTech Research*.

[27] Yüksel N., Karataş A., Baykara T. (2003). Comparative evaluation of granules made with different binders by a fluidized bed method. *Drug Development and Industrial Pharmacy*.

[28] Onunkwo G. C. (2010). Evaluation of okro gum as a binder in the formulation of thiamine hydrochloride granules and tablets. *Research in Pharmaceutical Biotechnology*.

[29] Wang C. G., Fang J. G. (2013). Pharmaceutical powders flow properties characterization: methods and applications. *Chinese Journal of New Drugs*.

[30] May R. K., Ke S., Han L. (2013). Hardness and density distributions of pharmaceutical tablets measured by terahertz pulsed imaging. *Journal of Pharmaceutical Sciences*.

[31] Saleem M., Shahin M., Srinivas B., Begu A. (2015). Evaluation of tablets by friability apparatus. *International Journal of Research in Pharmacy and Chemistry*.

[32] Jackson C., Akpabio E., Umoh R., Adedokun M., Ubulom P., Ekpe G. (2012). Evaluation of S*esamum indicum* gum as a binder in the formulation of paracetamol granules and tablets. *Research in Pharmaceutical Biotechnology*.

[33] Markl D., Zeitler J. A. (2017). A review of disintegration mechanisms and measurement techniques. *Pharmaceutical Research*.

[34] Eziuzo O. S., Amarauche C. (2017). Evaluation of the binding property of *Sida acuta* gum in paracetamol tablet formulations. *World Journal of Pharmaceutical Research*.

[35] Ghayas S., Sheraz M. A., Anjum F., Baig M. T. (2013). Factors influencing the dissolution testing of drugs. *Pakistan Journal of Medical Research*.

[36] Pant S., Sharma P. K., Malviya R. (2015). Evaluation of different concentration of binders on the dissolution profile of paracetamol tablets. *Advances in Biology Research*.

[37] USP 30/NF 25 (2007). *United States Pharmacopoeial*.

[38] Girhepunje K., Arulkumaran P. R., Maski N., Thirumoorthy N. (2009). A novel binding agent for pharmaceutical formulation from *Cassia roxburghii* seeds. *International Journal of Pharmacy and Pharmaceutical Sciences*.

